# The *vestigial* Quadrant Enhancer is dispensable for pattern formation and development of the *Drosophila* wing

**DOI:** 10.17912/micropub.biology.000585

**Published:** 2022-06-13

**Authors:** Keity J Farfán-Pira, Teresa I Martínez-Cuevas, Rosalio Reyes, Timothy A Evans, Marcos Nahmad

**Affiliations:** 1 Department of Physiology, Biophysics, and Neurosciences, Centre for Research and Advanced Studies of the National Polytechnic Institute (Cinvestav-IPN); 2 Department of Biological Sciences, University of Arkansas

## Abstract

In *Drosophila* , the pattern of the wing selector gene, *vestigial* ( *vg* ), is established by at least two enhancers: the Boundary Enhancer, which drives expression along the disc’s Dorsal-Ventral boundary; and the Quadrant Enhancer (QE) that patterns the rest of the wing pouch. Using CRISPR/Cas9 editing, we deleted DNA fragments around the reported QE sequence and found that the full Vg pattern is formed. Furthermore, adult wings arising from these gene-edited animals are normal in shape and pattern, but slightly smaller in size, although this reduction is not wing-specific in males. We suggest that other enhancers act redundantly to establish the *vg* pattern and rescue wing development.

**Figure 1. Vg patterning and adult wing development is largely unaffected in CRISPR-edited animals that lack the QE sequence.**
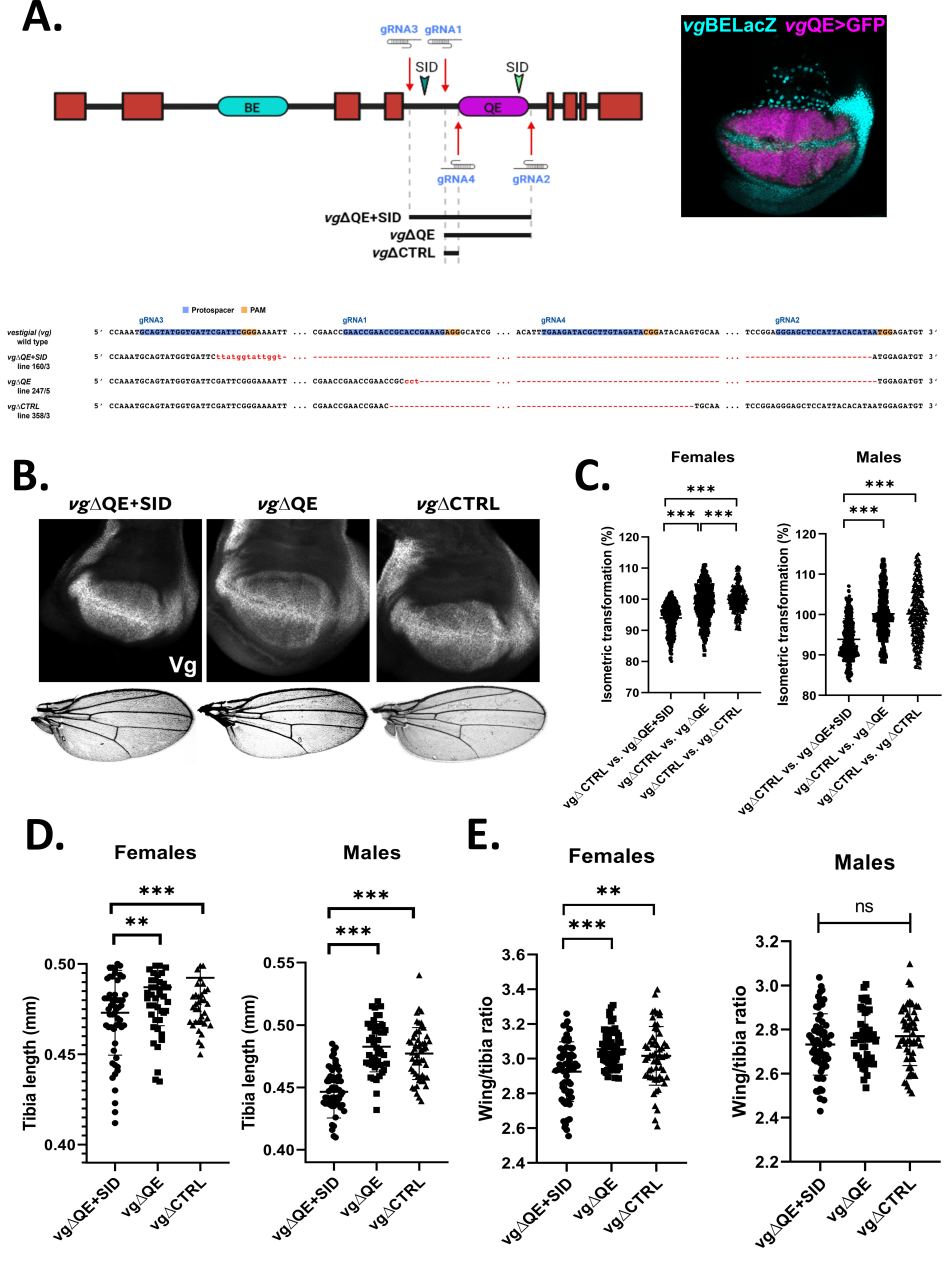
**(A)** Scheme of the *vg* gene showing the two intronic enhancers (BE, Boundary Enhancer; QE, Quadrant Enhancer) that account for the full Vg pattern in the third-instar wing imaginal disc (photo in the right: vgBELacZ=reporter of the BE marked by βGal immunostaining; vgQE>GFP=reporter of the QE marked by GFP expression under the Gal4-UAS system). The location of gRNAs (marked by red arrows) that were used to delete different fragments (black bars) within the fourth intron that contains the QE are shown: *vg* ∆QE+SID (gRNA3 and gRNA2, that contains an additional SID (green arrowhead) outside of the reported QE element), *vg* ∆QE (gRNA1 and gRNA2), or *vg* ∆CTRL. The sequencing results confirming each of the CRISPR/Cas9 deleted elements are shown; gRNAs protospacers (highlighted in blue) and PAM sites (highlighted in orange) are illustrated; sequence mismatches are displayed in red font. **(B)** Photos of representative wing imaginal discs displaying late third-instar larvae immunostained with Vg and female adult wings for each of the CRISPR/Cas9 edited lines. **(C)** Pairwise comparison of control *vs* . QE-deleted wings. An isometric transformation is applied to each control wing to match an experimental wing and the isometric transformation factor (in %) is plotted. Female and male wings are independently compared. Groups that are statistically significant after a one-way ANOVA analysis are shown. **(D, E)** Comparison of anterior tibia lengths (D) and wing to tibia length ratios (E) in each of the CRISPR/Cas9 edited lines. Female and male animals are independently compared. Groups that are statistically significant after a one-way ANOVA analysis are shown. (**:p-value<0.005; ***:p-value<0.0005; ns: not statistically-significant).

## Description

Activation of gene expression during development is controlled by enhancers, which are DNA sequences that contain transcription factor binding sites and drive the recruitment of the transcriptional machinery in a context-specific manner (Field & Adelman, 2020; Furlong & Levine, 2018; Long et al., 2016). Complex developmental patterns often require the action of one or multiple enhancers, which are precisely coordinated in space and time. Sometimes, two or more *cis* -regulatory elements establish an overlapping pattern of gene expression and they are referred as shadow enhancers (Hong et al., 2008). Shadow enhancers appear to be widespread in metazoan genomes suggesting that their role may be evolutionary conserved to confer robust gene expression patterns under genetic or environmental perturbations (Kvon et al., 2021).

In *Drosophila* , wing fate is determined by the expression of the selector gene, *vestigial* ( *vg* ), which is confined to an area within the wing imaginal disc referred as the wing pouch (Kim et al., 1996; Williams et al., 1991; Williams et al., 1993). The *vg* pattern is established by two intronic enhancers: the Margin or Boundary Enhancer (BE), and the Quadrant Enhancer (QE; Fig. 1A). Early in wing disc development, the BE drives *vg* expression in cells abutting the Dorsal-Ventral (DV) boundary (cyan staining, Fig. 1A) in response to a short-range Delta/Serrate-Notch and Wingless (Wg) signaling (Couso et al., 1995; de Celis et al., 1996; Doherty et al., 1996; Irvine & Vogt, 1997; Kim et al., 1995; Williams et al., 1994). As these Vg-expressing cells proliferate, they leave the DV signaling center, but maintain *vg* expression presumably through Polycomb/Trithorax Responsive Elements (PRE) and Vg autoregulation (Ahmad & Spens, 2019; Halder et al., 1998; Klein & Arias, 1999; Pérez et al., 2011; Simmonds et al., 1998). In addition, the QE drives *vg* expression in the rest of the wing pouch through the integration of several signaling networks including the long-range action of the Wg and Decapentaplegic (Dpp) morphogens (Kim et al., 1996; Klein & Arias, 1998; Lecuit & Cohen, 1998; Nellen et al., 1996; Neumann & Cohen, 1996; Zecca et al., 1996) and a Fat/Dachsous polarization signal that results in the recruitment of Vg in neighboring cells (Zecca & Struhl, 2007a; Zecca & Struhl, 2007b; Zecca & Struhl, 2010). At the transcriptional level, the recruitment signal depends on the nuclear translocation of Yorkie (Yki), the effector of the Warts-Hippo tumor suppressor pathway, which binds the TEAD-transcription factor Scalloped (Sd) and activate *vg* expression through Scalloped-Interaction Domains (SIDs) (Simmonds et al., 1998; Zecca & Struhl, 2010). A bioinformatic analysis reveals two SIDs within the fourth intron of the *vg* gene; one within the reported QE sequence (Klein & Arias, 1999; Williams et al., 1994; Zecca & Struhl, 2007a) and another one located 544 base pairs upstream of it (Fig. 1A).

Transgenic reporters show that the QE can drive *vg* expression in most of the wing pouch (magenta staining, Fig. 1A), but it is unknown whether or not the QE is necessary for *vg* expression and wing development. In order to investigate this, we designed guide RNAs (gRNAs) to delete the reported QE ( *vg* ∆QE; using gRNA1 and gRNA2, Fig. 1A) or the reported QE plus the additional SID ( *vg* ∆QE+SID; using gRNA3 and gRNA2, Fig. 1A) using CRISPR/Cas9 Non-Homologous End-Joining (NHEJ) technology (see Methods; Evans, 2017; Port et al., 2014). As a control, we deleted a small piece of DNA that does not overlap with the reported QE sequence nor contains a SID ( *vg* ∆CTL; using gRNA1 and gRNA4; Fig. 1A). However, none of these deletions have an effect in the Vg pattern of late third-larval wing discs, which appears to cover all the wing pouch, nor in the shape and pattern of the adult wing (Fig. 1B). We did notice a minor, albeit significant reduction in wing size of the *vg* ∆QE+SID line in both males and females with respect to the other deletions (Fig. 1C). Strikingly however, this reduction is not specific to the wing, as measurements of anterior legs’ tibias are also significantly smaller in *vg* ∆QE+SID animals with respect to the other lines, especially in males (Fig. 1D). In fact, when each wing measurement is normalized to the length of the corresponding tibia (wing to tibia ratio), we found that the size difference only persist in females (Fig. 1E), suggesting that *vg* ∆QE+SID males are proportionally smaller. These results highlight the potential impact of the additional SID on size control. Taken together, our data suggest that the QE is not required for Vg nor wing patterning, except for small sex-specific effects in animal size that occur only with an additional SID is deleted. However, we cannot rule out that Vg expression levels or its dynamics may be affected by the deletions.

Given the importance of Vg expression for wing differentiation, other regulatory elements may ensure that animals develop fully functional wings upon genetic mutations in the QE. This suggests the existence of shadow enhancers within the *Drosophila* genome that rescue the Vg pattern in the absence of the QE. These shadow enhancers likely respond to the same signaling pathways since the Vg pattern does not expand without these signals (Zecca & Struhl, 2007b). In addition, our data reveal the potential impact of SIDs on animal size. How could a regulatory sequence within the wing selector gene have an effect in the overall size of an animal? One possibility is that SIDs affect overall size through Yki-Sd binding, which is known to promote cell growth and/or proliferation in a plethora of systems (Goulev et al., 2008; Hariharan, 2015; Huang et al., 2005; Wu et al., 2008). Alternatively, perhaps the *vg* ∆QE+SID deletion affects size in a wing-specific manner and inter-organ coordination of size is established through systemic signals (Boulan et al., 2019; Colombani et al., 2012; Mesquita et al., 2010). Another interesting finding to explore in future work is the sex-specific wing *vs.* overall size difference (Fig. 1D,E). Since the *sd* gene is located in the first chromosome (Dmel\sd, FlyBase ID FBgn0003345), it is plausible that males and females respond differently to SID deletions. Finally, since *vg* is an evolutionary-conserved wing selector gene in insects (Abouheif & Wray, 2002; Clark-Hachtel et al., 2013; Clark-Hachtel et al., 2021; Zhang et al., 2021), our work highlights the impact of CRISPR/Cas9 editing to understand the contribution of regulatory elements to wing diversity (Medved et al., 2015; Linz & Tomoyasu, 2018).

## Methods

**gRNA Design**

Target sites were designed using flyCRISPR online tool CRISPR Optimal Target Finder (https://flycrispr.org) (Iseli et al., 2007; Gratz et al., 2014).

**Construction of gRNA plasmid**

Cloning was performed using pCFD4-U6:1_U6:3tandemgRNAs vector, that allows in-tandem expression of gRNA sequences (Port et al., 2014; Evans, 2017), through PCR products using pair of primers 1 and 2, 2 and 3, 1 and 4, and 3 and 4 (see Reagents), amplified with 2X Phusion Flash PCR Master Mix. Gibson Assembly was performed with PCR products and pCFD4 *Bbs* I digested vector. Clonings was confirmed by Sanger sequencing by Eurofins Genomics prior to injection.

**Identification of CRISPR-modified alleles**

The *vg ^QE^* gRNA plasmid was injected into *nos* -Cas9 embryos by Best Gene (Chino Hills, CA). Injected individuals (G0) were crossed as adults to *Sco/CyoRFP.* Founders (G0 flies producing F1 progeny carrying modified QE alleles) were identified using pools of three females derived from each G0 cross by PCR with primers 5 and 12 (for *vg* ∆QE+SID) or 12 and 13 (for *vg* ∆QE and *vg* ∆CTRL) which produce 0.5-kb or 1.5-kb respectively when the respective NHEJ (Non-Homologous End Joining) are present. From each identified founder, 5-10 F1 males were then crossed individually to *Sco/CyORFP* virgin females. After 3 days, F1 males were removed from the crosses and tested by PCR with the same set of primers to verify if they carried the modified allele. F2 flies from F1 crosses were used to generate balanced stocks, and the modified alleles were sequenced from genomic DNA using primers 12, 18 and 13 (see Reagents).

**Fly stocks and wing imaginal disc immunostaining**

The disc shown in Fig. 1A was obtained by crossing flies carrying a transgenic reporter of the vg BE in the second chromosome (vgBElacZ [obtained from Marco Milán]) with flies expressing nuclear GFP under the Gal4-UAS system (vgMQGal4 / SM6; UAS-GFP _nls_ / TM6B, Tb). For the immunostaining, wandering third-instar larvae were dissected in PEM (Na-Pipes 80mM + EGTA 5mM) solution. The internal tissue of anterior part was exposed with independent needles and larvae were fixed in 4% paraformaldehyde solution during 40 minutes at room temperature. Dissected larvae were then washed three times in PEM-T solution (PEM + Triton X100) in agitation for 10 minutes. Subsequently, samples were incubated with a blocking solution that contains PEM-T and 0.5% Bovine Serum Albumin (BSA), for two hours in agitation at room temperature. Blocking solution was discarded and primary antibodies were added (mouse anti-βGalactosidase at 1:1000 in Fig. 1A [no antibody was needed to detect GFP]; and Guinea-pig anti-Vg [kindly provided by Gary Struhl] at 1:200 in Fig. 1B) in staining solution (blocking solution + 1% Normal Goat Serum) and incubated overnight at 4 °C.

Primary antibodies were recovered, and the sample was washed three times with PEM-T solution at room temperature for 10 minutes. Incubations with secondary antibodies were performed with Alexa Fluor 647 anti-mouse (Fig. 1A) and Alexa Fluor 594 anti-Guinea Pig (Fig. 1B) at a concentration of 1:1000 each for two hours at room temperature in the dark. The sample was washed once again three times with PEM-T solution and one time with PEM solution for 10 minutes at room temperature in the dark. Discs were dissected and mounted using a stereoscopic microscope (Nikon SMZ800) in 15 µl of Mowiol solution.

**Confocal microscopy and image capture**

Imaginal wing disc micrographs were taken using a Spectral confocal microscope (Leica TCS-SP8) using a 63x objective (PL APO CS2, 63x, App. Num. 1.4 oil immersion) and the following specifications in LAS X software: Format 1024 x 1024, speed 600, Frame average 4, Phase X -33.48, Zoom factor 1.00, Z-step size 0.50, Smart gain 776.8 V, Smart offset -3.0% and Pinhole 1 AV. The images were stored in “.lif” format and subsequently analyzed with ImageJ software (https://imagej.nih.gov/ij/download.html) (Schneider et al., 2012). Values and statistical analysis were plotted in GraphPad Prism version 8.0.1.

**Isometric transformation**

Using Python (3.7.6), we developed a code that given two numpy arrays of the same length (referred with the sub-index 1 and 2):

Σ( *x*_1i_ -µ *x*_2i_ ) ^2^ + ( *y*_1i_ - *µy*_2i_ ) ^2^

where ( *x*_1i,_ *y*_1i_ ) are the coordinates of the i-th point of the standardized array 1 and µ is the contraction factor. These points are selected to be the intersection between each vein or intervein with each other or with the wing margin.

**Wing/tibia measures**

Adult flies of *vg* ∆QE+SID, *vg* ∆QE, and *vg* ∆CTRL lines were separated by sex, using a stereoscopic microscope (Nikon SMZ800) and preserved in 1ml 70% ethanol for dehydration for 12 hours. Each specimen was dissected in 15μl of 50% ethanol to obtain each pair of wings the corresponding pair of front legs mounted in microscope slides.

Wings and legs were photographed in a binocular microscope (Nikon Eclipse Ci) attached to a camera (ProgRes® CT5, Jenoptik) to allow measures (in wings, length of area between veins L3 and L4; in legs, length of tibia), using the ProgRes® Capture Pro-2.9 software. Measurements were performed using ImageJ software 1.53c (Wayne Rasband, National Institutes of Health, USA) using corresponding calibration for 4X objective (Distance in pixels: 100.501; known distance: 0.1; pixel aspect ratio: 1.0, unit of length: mm). Ratio of each wing to tibia proximal distal length were plotted using GraphPad Prism version 8.0.1.

**Statistical analysis**

A one-way ANOVA was performed to compare the wing to tibia ratios of each of the fly lines generated through CRISPR, with α =0.05. Subsequently, to establish the significant differences between groups, Tukey’s range test was performed with α = 0.05.

## Reagents

**Table:** 

**REAGENT**	**SOURCE**	**IDENTIFIER**
PIPES sodium salt	Sigma-Aldrich	Sigma Prod. No. P2949
EGTA Ethylene glycol-bis(2-amino-ethylether)-N,N,N’,N’-tetraacetic acid	Sigma-Aldrich	Sigma Prod. No. E3889
Triton X-100	Sigma-Aldrich	Sigma Prod. No. T9284
8% paraformaldehyde	Electron Microscopy Science	Cat. # 157-8
Bovine Serum Albumin BSA	Sigma-Aldrich	Sigma Prod. No. A2058
Normal Goat Serum	Invitrogen	Catalog # 31872

**Table:** 

**GENOTYPE**	**AVAILABLE FROM**
vgBElacZ (II)	Marco Milán
vgMQGal4;UAS-GFPnls	Bloomington Drosophila Stock Center, Stocks #8230 and #4776
*vg* ∆QE+SID	This study
*vg* ∆QE	This study
*vg* ∆CTRL	This study
yw;nos-Cas9(III-attP2)/TM6,Tb,Sb	NIG-FLY # CAS-0012
w; Sco/CyO-RFP	Generated in Evans laboratory

**Table:** 

**PLASMID**	**GENOTYPE**	**DESCRIPTION**
pCFD4	pCFD4-U6:1_U6:3tandemgRNAs	Addgene plasmid # 49411; http://n2t.net/addgene:49411 ; RRID: Addgene_49411

**Table:** 

**ANTIBODY**	**ANIMAL AND CLONALITY / SOURCE**	**DESCRIPTION**
Anti-Vestigial	Guinea pig polyclonal	Kindly provided by Gary Struhl (Columbia University)
Anti-β-Galactosidase	Mouse polyclonal, Promega	Catalog #Z378A
Alexa Fluor 594 goat anti-Guinea Pig IgG (H+L)	Thermo Fisher Scientific	Catalog # A-11076
Alexa Fluor 647 goat anti-Mouse IgG (H+L)	Thermo Fisher Scientific	Catalog #A-21236

**Table:** 

**OLIGO 5’- 3’**	**NAME**	**SEQUENCE**
**1**	**QE5’**	GGAAAGATATCCGGGTGAACTTCGAACCGAACCGCACCGAAAGGTTTTAGAGCTAGAAATAGCAAG
**2**	**QE3’**	GCTATTTCTAGCTCTAAAACTTATGTGTAATGGAGCTCCCGACGTTAAATTGAAAATAGGTC
**3**	**TEA2**	GGAAAGATATCCGGGTGAACTTCGCAGTATGGTGATTCGATTCGTTTTAGAGCTAGAAATAGCAAG
**4**	**Neg**	GCTATTTCTAGCTCTAAAACTATCTACAAGCGTATCTTCCGACGTTAAATTGAAAATAGGTC
**5**	**LHA1- F**	ACATGCATGCATGTGGAAATGCCACCACTTTGTGCG
**12**	**RHA3-R**	CCGCTCGAGGAAATCGCGCGACGCCGCC
**13**	**LHA RecSp-F**	CGCGGATCCCTAGTTGGAATGTGCTAT
**18**	**colLHA2-F**	GCTGCTCGAAAATAACTGGG
